# An update on novel taxa and revised taxonomic status of bacteria isolated from human clinical specimens described in 2024

**DOI:** 10.1128/jcm.01068-25

**Published:** 2025-10-17

**Authors:** Arianna Carella, Karen C. Carroll, Erik Munson

**Affiliations:** 1 Department of Medical Laboratory Science, Marquette University 5505, Milwaukee, Wisconsin, USA; 2 Division of Medical Microbiology, Department of Pathology, The Johns Hopkins University School of Medicine 1500, Baltimore, Maryland, USA; 3 Wisconsin Clinical Laboratory Network Laboratory Technical Advisory Group, Madison, Wisconsin, USA; Medical College of Wisconsin, Milwaukee, Wisconsin, USA

**Keywords:** taxonomy, nomenclature, prokaryotes

## Abstract

Knowledge of novel and revised bacterial taxonomy facilitates routine operations in the medical microbiology laboratory and communication with important stakeholders. Notable new gram-positive taxa accepted in 2024 include the clinically significant *Staphylococcus brunensis* sp. nov., as well as *Streptococcus suis* subsp. *hashimotonensis* subsp. nov., which can be delineated from other *Streptococcus suis* subsp. by possession of the Lancefield group A antigen. Four novel *Enterobacterales* species are discussed, plus a novel eighth family *Gallaecimonadaceae*. One of these, *Providencia huashanensis* sp. nov. is clinically significant and harbors multiple genetic determinants that encode antimicrobial resistance mechanisms. *Stenotrophomonas forensis* sp. nov., derived from specimen transport medium, was determined to be a distinct component within the *Stenotrophomonas maltophilia* complex. *Bartonella tamiae* sp. nov. has become officially recognized as the agent of the first culture-confirmed bartonelloses in Thailand. Significant taxonomic revisions include the return of *Chlamydophila caviae* to the *Chlamydia* genus, creation of the novel *Metaclostridioides* genus for *Clostridioides mangenotii*, and reclassification of two *Kocuria* spp. into two subspecies of *Kocuria rosea*. Clinical updates are provided for 13 past *Journal of Clinical Microbiology* taxonomic compendium entries for which extensive clinical data were not previously available. These include potential clinical significance for non-*vaginalis* species of the *Gardnerella* genus and for *Pandoraea commovens* in a non-cystic fibrosis setting.

## INTRODUCTION

The long-standing goal of the *Journal of Clinical Microbiology* publication series on novel and revised prokaryotic taxonomy relative to human primary specimens is to provide data that clinical microbiology laboratorians can synthesize and integrate, if need be, into routine clinical laboratory practice. Discussion intends to be limited to this overarching objective, as well as provision of additional clinical information (if found in primary publication) relative to major revisions and potentially highly significant organisms. Authors of past reviews summarizing novel taxa and revisions to taxonomy relative to the virology (1), parasitology (2), mycobacteriology (3), mycology (4), and bacteriology (5) of human clinical specimens serve merely as informants, rather than influencers. The publication of bacterial taxonomy reviews on an annual basis for the past 2 years (5, 6) has become consistent with a recent Clinical and Laboratory Standards Institute (CLSI) guideline (7) for implementation of important novel and revised taxonomy into routine clinical laboratory practice.

Advances in whole genome and protein sequencing, as applied to the human microbiome, continue to result in the expansion of novel taxa. Contributions of additional isolates toward pre-existing names often signal a need to reclassify existing taxa. As more becomes known about the microbiome of particular human anatomic sites, the role of microbiota in health and disease associations is elucidated. Moreover, early investigations into the role that select bacteria can play in non-infectious conditions can be facilitated. A total of 52 nomenclatural elements of interest (40 novel taxa and 12 taxonomic revisions) relative to human clinical material published in 2024 are presented in the following review.

## MATERIALS AND METHODS

Effectively described taxa are those novel microbial species that have undergone extensive polyphasic characterization, possess a representative type strain, and have been demonstrated not to possess a synonym taxon. Per the International Committee on Systematics of Prokaryotes, validly published novel and revised taxa pertinent to prokaryotic species must satisfy requirements as set forth by the International Code of Nomenclature of Prokaryotes (ICNP) (8). Formal validation of a taxon makes the name available for use in the nomenclature of domains *Bacteria* and *Archaea*. Without such validation, a designation may exist in the literature as an effective publication but would not be recommended for use if following the rules of the ICNP.

One fulfillment for valid publication involves a properly conscripted Latin binomial name and descriptive features of a novel species being published in the *International Journal of Systematic and Evolutionary Microbiology* (*IJSEM*). Required information must also include proof of deposit of type strains into recognized cultured collections located in two separate nations. An example relative to the gram-positive coccus *Aerococcus loyolae* is provided in reference 9. As of January 2018, taxon descriptions in *IJSEM* require that description of the 16S rRNA gene (or other taxonomically relevant genes) sequence accession number relative to the type strain be deposited in GenBank (10), with genome accession number included as part of the effective description. The similarity of this sequence is to be assessed versus related taxa.

As an alternative to primary publication in *IJSEM*, studies may be published in another journal and later included on the Validation List in *IJSEM* if specific requirements for valid publication of new names have been met. One past example relative to bacteria derived from clinical material is the effective description of the allegedly motile, gram-negative bacillus *Acinetobacter ihumi* sp. nov. (11), with subsequent inclusion on an *IJSEM* Validation List (12). To be considered for inclusion on a Validation List, which is published six times per year, authors must submit a copy of the previously published manuscript to the editorial office of *IJSEM* for confirmation that all elements necessary for valid publication (including culture collections) have been met. Approximately one-half of effectively described, but non-*IJSEM*-published, taxa are not submitted to *IJSEM* for inclusion on Validation Lists (13).

Non-*IJSEM* journals that have recently published studies providing a valid description of human-derived novel taxa pertinent to this report, which may be relevant to the practice of medical microbiology, include *Antonie Van Leeuwenhoek*, *Archives of Microbiology*, *Current Microbiology*, *Frontiers in Cellular and Infection Microbiology*, *International Journal of Antimicrobial Agents*, *Journal of Clinical Microbiology*, *Microbiology Spectrum*, *New Microbes and New Infections*, *Research in Microbiology*, *Scientific Reports*, *Standards in Genomic Sciences*, *Systematic and Applied Microbiology*, and *Vector-Borne Zoonotic Diseases*. Journals that have recently published studies reflecting revisions in prokaryotic taxonomy relative to human clinical material include *BMC Genomics* and *Systematic and Applied Microbiology*.

A taxon validly published following an effective description in *IJSEM* or by inclusion on a Validation List may later be identified as a synonym (i.e., ascertainment of an identical organism with an antecedent taxonomic designation). Moreover, Trujillo et al. (14) stated that once a taxon is validly published or included on a Validation List, it remains available for use as long as both it is the correct name for the appropriate circumscription, position, and rank and it is not subsequently rejected (*nomina rejicienda*) through a formal rejection process in accordance with ICNP. Organisms that are transferred to another taxon frequently do not relinquish a validly published status; the majority of prokaryotic nomenclature revisions are due to changes in taxonomic opinion. As an example, the gram-negative diplococcus *Branhamella catarrhalis* (15) currently remains validly (and effectively) published as a synonym to its antecedent (and correct) name, *Moraxella catarrhalis* (16). Table headers throughout this document include phrases such as “Earlier validly published name” and “2024 validly published name” to reflect this paradigm.

All issues of *IJSEM* published from January 2024 to December 2024 (including six Validation Lists) were manually searched for original articles describing novel, validly published species taxonomy or accepted changes in taxonomic nomenclature. With the goal of consistency, only nomenclature with taxonomic status of “correct name” (per data curated by List of Prokaryotic names with Standing in Nomenclature [LPSN], https://lpsn.dsmz.de) was included. On rare occasions, taxa accepted in *IJSEM* during calendar year 2024 (e.g., the gram-positive bacterium *Rhodococcoides fascians* comb. nov. publication in reference 17 and inclusion on Validation List no. 216 [18]) were subsequently classified by LPSN as synonyms (e.g., correct name *Rhodococcus fascians* [19]) and were thus excluded. The audit was further filtered by organisms recovered from human clinical material. When an initial organism reservoir could not be ascertained, PubMed primary literature searches (US National Library of Medicine and the National Institutes of Health) attempted to index subsequent literature for further investigation; a number of these supplemental reports are referenced throughout this review.

Some primary publications simply identified isolates as being derived from a specific specimen source (including traditional sterile body sites) but did not provide contextual clinical data. In such scenarios, the clinical significance of these taxa was interpreted as “not established.” Moreover, a number of novel taxa included within this compendium (20, 21), including *Enterobacter pasteurii* sp. nov., were derived from blood cultures but were not accompanied by any summary of clinical manifestations of infection. In two novel *Streptococcus* spp. publications (22, 23), potential anatomic sources of infection were presented that could also be inhabited by commensal flora. Within these descriptions, no clinical data were presented (including clinical signs and symptoms and/or laboratory data that could suggest an acute inflammatory process). These paucities of provided clinical data relegate pathogenicity and clinical significance assessments of these isolates to debate. Additional studies and published case reports (24, 25) from reliable journal outlets (26) can assist in the further clinical characterization of novel taxa.

## RESULTS AND DISCUSSION

A compilation of novel taxa recovered from human sources stratified by Gram morphology and oxygen growth requirement (when fully provided) is presented in Table 1, with the phenotypic means of taxa organization within Table 1 designed with the practicing medical microbiologist in mind. Organisms are presented in alphabetical order (rather than chronological order) within each stratification. Table 2 provides taxonomic revisions for organisms originally recovered from human sources. On the basis of recent peer-reviewed publications, Table 3 attempts to retrospectively ascribe clinical relevance to a number of taxa whose significance was inconclusive in previous *Journal of Clinical Microbiology* compendia (5, 27–30). Findings that warrant emphasis are discussed below.

**TABLE 1:** Novel, validly published bacterial species recovered from human clinical material reported from January 2024 to December 2024

Scientific name	Family	Source^*a*^	Clinical relevance	Growth characteristics^*h*^	References
Gram-positive cocci					
*Dolosigranulum savutiense* sp. nov.	*Carnobacteriaceae*	Isolates (4) from nasopharyngeal swabs collected from mother-infant dyads in Botswana	Not established	Facultative, non-motile, catalase-negative, oxidase-negative, non-spore-forming gram-positive coccus; small, gray/white, flat, smooth colonies observed on sheep blood agar; variable α-hemolysis; optimal growth at 37°C in 5% CO_2_ environment; positive for hippurate, pyrrolidonyl arylamidase; three strains positive for leucine aminopeptidase; negative for β-galactosidase, L-arabinose, D-ribose, trehalose, inulin, raffinose; nonadecanoic acid, 2-hydroxypentadecanoic acid utilization exclusively observed in *D. savutiense* sp. nov. and differentiated from *Dolosigranulum pigrum*; growth inhibition noted (no interpretive criteria) with penicillin, vancomycin, tetracycline, levofloxacin, clindamycin, erythromycin, and gentamicin	(31)
*Staphylococcus brunensis* sp. nov.	*Staphylococcaceae*	Isolates from ear (2), bile (1), and wound pus (2) from Czech Republic	Otitis media noted in two patients; co-cultured with other pathogenic agents in wound infection and cholecystitis	Aerobic, non-motile, catalase-positive (80% of isolates), oxidase-negative, non-spore-forming gram-positive coccus arranged in pairs, tetrads, clusters; 2 mm diameter circular, flat, smooth, shiny, white colonies on sheep blood agar; growth in the presence of 12% NaCl at 20°C and 45°C, but not at 15°C and 48°C; positive for arginine dihydrolase, Voges-Proskauer, β-glucuronidase, lactose, galactose; negative for urease, DNase, mannose, ribose, *N*-acetylglucosamine, D-arabinose; susceptible to cefoxitin, clindamycin, gentamicin, oxacillin, linezolid, trimethoprim/sulfamethoxazole, and tetracycline; intermediate susceptibility to ciprofloxacin and levofloxacin; strain-dependent resistance to penicillin G and erythromycin	(32)^*b*^
*Streptococcus chosunensis* sp. nov.	*Streptococcaceae*	Post-operative maxillary cyst lesion from a male in the Republic of Korea	No additional clinical data provided	Originally published as *Streptococcus chosunense* sp. nov.; facultative, non-motile, non-spore-forming gram-positive coccus; 0.5 mm diameter opaque, convex, smooth, circular, white, entire colonies on trypticase peptone medium supplemented with yeast extract; demonstrated α-hemolysis on sheep blood agar; optimal growth at 37°C; positive for β-galactosidase, lactose, and starch; negative for arginine dihydrolase, α-galactosidase, raffinose, ribose, sorbitol, and mannitol	(22)^*c*^
*Streptococcus gwangjuensis* sp. nov.	*Streptococcaceae*	Human pericoronitis lesion in the Republic of Korea	No additional clinical data provided	Originally published as *Streptococcus gwangjuense* sp. nov.; facultative gram-positive coccus; 0.4 mm diameter white, opaque, convex colonies on BHI agar; demonstrated α-hemolysis on BHI agar containing 5% sheep blood; optimal growth at 37°C–40°C; positive for β-galactosidase, leucine aminopeptidase, and lactose; negative for arginine dihydrolase, esculin, hippurate, α-galactosidase, mannitol, raffinose, sorbitol, and trehalose	(23)^*c*^
*Streptococcus hohhotensis* sp. nov.	*Streptococcaceae*	Breast milk from a nursing mother in China	Not established	Facultative, catalase-negative, oxidase-negative gram-positive coccus; 0.2- to 0.3 mm diameter white, opaque, round, raised, smooth α-hemolytic colonies observed on M17 agar; optimal growth at 37°C; positive for α-glucosidase, lactose, raffinose, alkaline phosphatase, salicin, melibiose, sucrose, and *N*-acetyl-β-glucosaminidase; negative for D-ribose and D-mannose; susceptible to linezolid; resistant to clindamycin, tetracycline, ampicillin, erythromycin, and chloramphenicol	(33)
*Streptococcus humanilactis* sp. nov.	*Streptococcaceae*	Isolates (2) from breast milk of healthy nursing mothers in China	Not established	Facultative, catalase-negative gram-positive coccus; 0.4 mm diameter white, opaque, raised, rounded colonies on M17 agar; α-hemolytic on media containing sheep blood; optimal growth at 37°C–40°C; no growth in NaCl; positive for α-galactosidase, β-glucosidase, raffinose, amidon, alkaline phosphatase, valine arylamidase, and acid phosphatase; negative for β-galactosidase, arginine dihydrolase, ribose, D-mannose, salicin, and D-melibiose	(34)^*c*^
*Streptococcus* *raffinosi* sp. nov.	*Streptococcaceae*	Isolates (3) from breast milk of healthy females in Vietnam	Not established	Facultative, catalase-negative, non-spore-forming gram-positive coccus; 0.4–0.8 mm diameter round, convex, cream-colored colonies on deMan–Rogosa–Sharpe agar; α-hemolytic colonies on Columbia agar with 5% sheep blood; optimal growth at 37°C; growth in 2% NaCl; positive for acetoin, esculin; positive for β-galactosidase, α-galactosidase, urease, lactose, raffinose, maltose, and melibiose; negative for hippurate, β-glucosidase, trehalose, and methyl-β-D-glucopyranoside; *ermB* detected	(35)
*Streptococcus suis* subsp. *hashimotonensis* subsp. nov.	*Streptococcaceae*	Wound infection of a human bitten by a boar in Japan	Admitted for fever, chills, and purulent wound	Facultative, non-spore-forming gram-positive coccus; 1.2- to 1.5 mm diameter α-hemolytic (developing into β-hemolysis over time), convex, circular colonies on sheep blood agar; growth at 30°C–42°C; does not tolerate 6.5% NaCl; positive for Lancefield group A antigen, acid phosphatase, D-amygdalin, α-glucosidase, β-glucosidase, β-glucuronidase, α-chymotrypsin, and tagatose; negative for α-galactosidase, pyroglutamic acid arylamidase, and *N*-acetyl-β-glucosamidase; susceptible to bacitracin	(36)^*d*^
Gram-positive bacilli					
*Bacillus basilensis* sp. nov.	*Bacillaceae*	Superficial skin swab	Not established, isolate originally identified as *Bacillus cereus* or *Bacillus* genomospecies 13 by MALDI-TOF MS platforms	Aerobic gram-positive bacillus; gray, matte β-hemolytic (double zone) colonies on Columbia agar with 5% sheep blood at 37°C; positive for 6.5% NaCl and *N*-acetyl-D-glucosamine; negative for lysine arylamidase, maltotriose, esculin, pyruvate, alanine arylamidase, and phenylalanine arylamidase; susceptible to clindamycin and vancomycin; intermediate susceptibility to levofloxacin; resistant to penicillin	(37)^*b*^
*Cellulomonas endometrii* sp. nov.	*Cellulomonadaceae*	Endometrial biopsy from a patient in France	Recurrent early miscarriage and endometriosis	Facultative, motile (sliding), catalase-positive, oxidase-negative, non-spore-forming gram-positive bacillus; 2 mm diameter circular, pale yellow, opaque, convex colonies on Columbia agar with 5% sheep blood; optimal growth at 37°C; positive for β-galactosidase, lactose, D-sorbitol, D-raffinose, L-rhamnose, L-fucose, D-tagatose, *N*-acetylglucosamine, and D-glucose; negative for D-sucrose and D-mannose; reported MIC of 3 µg/L to amoxicillin, 2 µg/L to ceftriaxone, 0.038 µg/L to imipenem, 8 µg/L to ciprofloxacin, 8 µg/L to clindamycin, 0.023 µg/L to doxycycline, and 0.125 µg/L to vancomycin; resistant to amikacin, fosfomycin, metronidazole, and trimethoprim-sulfamethoxazole	(38)^*c*^
*Corynebacterium ihumii* sp. nov.	*Corynebacteriaceae*	Stool from a male patient admitted to intensive care for respiratory distress in France	Not established	Facultative, non-motile, catalase-positive, oxidase-negative, non-spore-forming gram-positive bacillus; 0.5 mm diameter white, granular colonies on blood-enriched Columbia agar; optimal growth at 37°C; positive for acid phosphatase, valine arylamidase, D-xylose, D-fructose, and D-mannose; negative for esterase, esterase lipase, cystine arylamidase, and 5-keto-gluconate; susceptible to amoxicillin, amoxicillin-clavulanic acid, ceftriaxone, imipenem, doxycycline, vancomycin, erythromycin, trimethoprim/sulfamethoxazole, and ciprofloxacin; resistant to metronidazole	(39)^*c*^
*Nocardia implantans* sp. nov.	*Nocardiaceae*	Isolates (2) from sputa of a patient in China	Pulmonary infection	Aerobic, non-motile, catalase-positive gram-positive bacillus; round, rough, dry, white colonies on BHI agar with 5% sheep blood; optimal growth at 37°C; growth partially inhibited by potassium tellurite; positive for L-rhamnose and β-glucosidase; weakly positive for β-galactosidase, D-fructose, amygdalin, salicin, and sucrose; negative for D-arabinose; intermediate susceptibility to moxifloxacin, cefoxitin, doxycycline, ciprofloxacin, and minocycline; resistant to amoxicillin-clavulanic acid and tigecycline	(40)
Gram-negative bacilli and coccobacilli					
*Bartonella tamiae* sp. nov.	*Bartonellaceae*	Isolates (3) from blood of patients in Thailand	Six-day fever and maculopapular rash in one patient; ocular pterygia in a second patient; fever, fatigue, myalgia, headache, and petechial rash in a third patient	Gram-negative bacillus recovered from culture in pre-enrichment cells or liquid growth medium with subculture onto rabbit blood agar (all modalities incubated in 35°C 5% CO_2_); positive for 3-indoxylphosphate; negative for *o*-nitrophenyl-β-D-glucopyranoside, *p*-nitrophenyl-α-L-fucopyranoside, *p*-nitrophenyl-α-D-mannopyranoside, L-proline-β-naphthylamide, trehalose, urea, indole, and nitrate; reported MIC of 4 µg/mL to doxycycline, >4 µg/mL to erythromycin, 0.5 µg/mL to gentamicin, and >4 µg/mL to cefotaxime	(41)^*d*^
*Enterobacter* *pasteurii* sp. nov.	*Enterobacteriaceae*	Human isolates (2) archived in reference collections in France, one known bacteremic patient	Not established	Facultative, non-motile, oxidase-negative, catalase-positive gram-negative bacillus (up to 20 µm filaments observed); circular, translucent, raised colonies with irregular margins on tryptic soy agar; optimal growth at 37°C; positive for Voges-Proskauer, citrate, β-galactosidase, D-mannitol, inositol, D-sorbitol, L-rhamnose, sucrose, meliobiose, and arabinose; negative for indole, urease, amygdalin, L-fucose, D-arabitol, dulcitol, and D-turanose; susceptible to ampicillin, piperacillin, cephalothin, ceftazidime, imipenem, ciprofloxacin, aztreonam, amikacin; resistant to erythromycin, pristinamycin, and fosfomycin	(20)^*e*^
*Enterobacter pseudoroggenkampii* sp. nov.	*Enterobacteriaceae*	Isolates (2) derived from pus and sputum specimens of hospitalized patients in China	One patient with diabetic foot ulcer, another with pneumonia	Facultative, motile, oxidase-negative, catalase-positive, non-spore-forming gram-negative bacillus; white, translucent, moist, bulging, round colonies on nutrient agar; optimal growth at 30°C–37°C; positive for β-galactosidase, arginine dihydrolase, ornithine decarboxylase, gelatinase, Voges-Proskauer, and gelatinase; negative for lysine decarboxylase, urease, indole, L-rhamnose, and H_2_S; susceptible to gentamicin, imipenem, ticarcillin, ceftazidime-avibactam, trimethoprim-sulfamethoxazole; resistant to ampicillin, ampicillin-sulbactam, cefazolin, cefuroxime, and colistin; variable susceptibility to ciprofloxacin, cefepime, aztreonam, ceftriaxone, piperacillin-tazobactam, and moxifloxacin	(42)^*b*^
*Kingella pumchi* sp. nov.	*Neisseriaceae*	Tissue from China	Vertebral puncture tissue of a hospitalized patient	Aerobic, non-motile, oxidase-positive, catalase-negative, non-spore-forming gram-negative bacillus; 1.5- to 2.0 mm circular, white, moist, opaque colonies on blood agar; optimal growth at 37°C; tolerates pH 8.0; positive for glucose, lipase, proline arylamidase, γ-glutamyl transferase, C4 esterase, and trypsin; negative for fructose, maltose, sucrose, ornithine decarboxylase, alkaline phosphatase, C8 esterase lipase, and acid phosphatase; susceptibility to penicillin, ceftriaxone, cefixime, tetracycline, and ciprofloxacin predicted by genetic characterization	(43)^*f*^
*Ottowia cancrivicina* sp. nov.	*Comamonadaceae*	Precancerous tissue from a patient’s stomach in China	Not established	Facultative, non-motile, oxidase-positive, catalase-positive gram-negative bacillus; beige, circular, entire colonies on nutrient agar; optimal growth at 37°C; capable of growth at 43°C; positive for gelatinase, C8 esterase lipase, valine arylamidase, *N*-acetyl-β-glucosaminidase, D-fructose, melezitose, and D-fucose; negative for nitrate, trypsin, D-arabinose, D-adonitol, L-rhamnose, inositol, amygdalin, arbutin, trehalose, and L-fucose; reported susceptibility to tetracycline, polymyxin B, and vancomycin; reported resistance to erythromycin, tobramycin, ampicillin, cefotaxime, lincomycin, ceftriaxone, clarithromycin, and ofloxacin	(44)
*Providencia huashanensis* sp. nov.	*Morganellaceae*	Abdominal drainage, urine, and cerebrospinal fluid from separate patients in China	Nosocomial incision infection, urinary tract infection, and intracranial infection	Facultative, non-motile, oxidase-negative, catalase-positive, non-spore-forming gram-negative bacillus; white, opaque, convex, circular, shiny colonies on Columbia blood agar; optimal growth at 35°C–37°C; positive for urease, D-mannitol, arbutin, esculin, glycerol, and salicin; negative for citrate, indole, gelatinase, raffinose, D-xylose, L-arabinose, L-rhamnose, cellobiose, sorbitol, and sucrose; susceptible to tigecycline; strain-dependent susceptibility to aztreonam-avibactam; resistant to colistin, amikacin, aztreonam, ciprofloxacin, ceftazidime, ceftazidime-avibactam, imipenem, imipenem-relebactam, and trimethoprim-sulfamethoxazole	(45)^*d*^
*Ralstonia flaminis* sp. nov.	*Burkholderiaceae*	Sputum from a cystic fibrosis patient in Canada	Not established	Aerobic, oxidase-positive, catalase-positive gram-negative bacillus; opaque, circular, convex, smooth colonies with wavy or smooth margins on tryptone soya agar; optimal growth between 15°C and 40°C; hemolysis on horse blood; growth on MacConkey, Drigalski, and Reasoner’s 2A (R2A) agars; no growth on cetrimide agar; positive for gelatin and casein; negative for starch, nitrate, and D-mannitol; susceptibility to trimethoprim-sulfamethoxazole, tigecycline, and cefepime; resistance to ceftazidime, colistin, aztreonam, ceftolozane-tazobactam, and amikacin	(21)^*c*^
*Ralstonia flatus* sp. nov.	*Burkholderiaceae*	Isolates (2) from blood and cystic fibrosis sputum in the United States	Not established	Aerobic, oxidase-positive, catalase-positive gram-negative bacillus; opaque, circular, convex, smooth colonies with wavy or smooth margins on tryptone soya agar; optimal growth between 15°C and 40°C; hemolysis on horse blood; growth on MacConkey, Drigalski, and R2A agars; no growth on cetrimide agar; positive for gelatin and casein; negative for starch, nitrate, and D-mannitol; susceptibility to trimethoprim-sulfamethoxazole; resistance to ceftazidime, colistin, aztreonam, ceftolozane-tazobactam, meropenem, and piperacillin-tazobactam; variable susceptibility to amikacin	(21)^*c*^
*Ralstonia holmesii* sp. nov.	*Burkholderiaceae*	Isolates (3) from the United Kingdom and the United States, including one from US tracheotomy	Not established	Aerobic, oxidase-positive, catalase-positive gram-negative bacillus; opaque, circular, convex, smooth colonies with wavy or smooth margins on tryptone soya agar; optimal growth between 15°C and 40°C; hemolysis on horse blood; growth on MacConkey, Drigalski, cetrimide, and R2A agars; positive for gelatin, casein, D-mannitol, and nitrate; negative for starch; susceptibility to trimethoprim-sulfamethoxazole and tigecycline; resistance to ceftazidime, colistin, aztreonam, ceftolozane-tazobactam, meropenem, piperacillin-tazobactam, and amikacin	(21)^*c*^
*Ralstonia thomasii* sp. nov.	*Burkholderiaceae*	Isolates (5) known to be derived from humans in Sweden, the United States, and United Kingdom; sources include blood and sputum	Not established	Aerobic, oxidase-positive, catalase-positive gram-negative bacillus; opaque, circular, convex, smooth colonies with wavy or smooth margins on tryptone soya agar; optimal growth between 15°C and 40°C; hemolysis on horse blood; growth on MacConkey, Drigalski, cetrimide, and R2A agars; positive for gelatin, nitrate; negative for starch, casein, and D-mannitol; susceptibility to trimethoprim-sulfamethoxazole, tigecycline, cefepime, and piperacillin-tazobactam; resistance to ceftazidime, colistin, aztreonam, ceftolozane-tazobactam, and meropenem; variable susceptibility to amikacin	(21)^*c*^
*Serratia sarumanii* sp. nov.	*Yersiniaceae*	Isolates from wound (3) and urine (1) specimens in Germany	Patients had elevated acute-phase reactant levels and leukocytosis; co-isolation of *Pseudomonas aeruginosa* and *Klebsiella oxytoca* from select specimens	Gram-negative bacillus; white colonies on solid Luria–Bertani agar; optimal growth at 30°C–38°C; non-hemolytic on 5% sheep blood agar; colorless colonies on MacConkey agar; growth in 7.5% NaCl; positive for α-glucosidase, β-galactosidase, D-maltose, L-lactate alkalinization, phosphatase, α-glucosidase, and β-*N*-acetyl-galactosaminidase; negative for urease, γ-glutamyl-transferase, lipase, palatinose, tyrosine arylamidase, and malonate; susceptible to gentamicin, ciprofloxacin, ertapenem, and trimethoprim/sulfamethoxazole; resistant to ampicillin, piperacillin, cefotaxime, and piperacillin/tazobactam	(46)^*d*^
*Stenotrophomonas forensis* sp. nov.	*Lysobacteraceae*	Tracheal aspirate, sputum, wound, bronchoalveolar lavage, tissue, and blood	Novel taxon within *Stenotrophomonas maltophilia* complex reflects human isolates previously classified as *S. maltophilia* or *Stenotrophomonas* spp.	Aerobic, non-motile gram-negative bacillus; 1.5- to 2.0 mm smooth, non-hemolytic colonies on trypticase soy agar with 5% sheep blood agar; optimal growth at 35°C; non-lactose-fermentative colonies on MacConkey agar; positive for β- *N*-acetyl-glucosaminidase, β-glucosidase, lipase, sodium citrate, L-lactate alkalinization, and succinate alkalinization; negative for maltose and malonate	(47)
*Stenotrophomonas pigmentata* sp. nov.	*Lysobacteraceae*	Patient with pulmonary tuberculosis in China (anatomic site not specified)	Not established, pathogenicity elucidated in a murine model	Aerobic, motile (single extreme flagellum), catalase-positive, non-spore-forming gram-negative bacillus; translucent, smooth, moist colonies with brown water-soluble pigment on nutrient agar; grayish-white, hemolytic colonies with ammonia odor on 5% sheep blood agar; growth observed at 15°C–37°C; positive for nitrate, gelatin, alkaline phosphatase, esterase lipase, leucine, and starch; negative for glucose and mannose; reported resistance to β-lactam agents, carbapenems, and trimethoprim-sulfamethoxazole (CLSI M24 cited for interpretation)	(48)^*d*^
Gram-positive anaerobes					
*Anaerolentibacter hominis* gen. nov., sp. nov.	*Lachnospiraceae*	Feces from a healthy donor in China	Not established	Anaerobic, non-motile gram-positive bacillus; 0.3- to 0.5 mm diameter smooth, wet, white, circular, entire colonies on modified Gifu anaerobic medium; optimal growth at 37°C; positive for alkaline phosphatase, C4 esterase, cellobiose, and acid phosphatase; negative for C8 esterase, α-galactosidase, β-galactosidase, β-glucuronidase, and β-glucosidase	(49)
*Collinsella acetigenes* sp. nov.	*Coriobacteriaceae*	Feces from a healthy human in the Republic of Korea	Not established	Anaerobic, non-motile, catalase-negative, oxidase-negative Gram-positive bacillus; <1 mm diameter creamy-white, circular colonies on Gifu anaerobic medium; optimal growth at 37°C; positive for leucine arylamidase, *N*-acetyl-β-glucosaminidase, α-fucosidase, acid phosphatase; negative for D-glucose, D-lactose, D-saccharose, D-maltose, D-mannose	(50)^*g*^
*Diplocloster hominis* sp. nov.	*Lachnospiraceae*	Feces from a healthy donor in China	Not established	anaerobic, motile Gram-positive bacillus; 1- to 2 mm diameter irregular, wet, yellow, convex, opaque colonies on modified Gifu anaerobic medium; optimal growth at 37°C; positive for alkaline phosphatase, C4 esterase, cellobiose, acid phosphatase, C8 esterase, β-glucuronidase; negative for α-galactosidase, β-galactosidase, β-glucosidase	(49)
*Eubacterium album* sp. nov.	*Eubacteriaceae*	Feces from a healthy female in China	Not established	anaerobic, non-motile, non-spore-forming Gram-positive bacillus; round, milk-white, slightly raised, wet colonies on Gifu anaerobic medium; optimal growth at 37°C; positive for raffinose, L-rhamnose, α-glucosidase, β-glucosidase; negative for cellobiose, maltose, D-mannose, alkaline phosphatase, esterase lipase, β-galactosidase, β-glucuronidase; reported susceptibility to chloramphenicol, penicillin, ciprofloxacin, clindamycin, ceftriaxone, vancomycin	(51)
*Faecalimicrobium dakarense* gen. nov., sp. nov.	*Peptostreptococcaceae*	Feces from a child in Senegal with gastroenteritis	Not established	Previous classification of *Clostridium dakarense* (52) never validly published; anaerobic, motile, catalase-negative, oxidase-negative, spore-forming gram-positive bacillus; 1.5 mm diameter opaque, smooth colonies on blood-enriched Columbia agar; optimal growth at 37°C; positive for acid phosphatase, D-glucose, D-maltose; negative for nitrate, β-galactosidase, mannose, mannitol, sucrose, and D-fructose; reported susceptibility to amoxicillin, metronidazole, vancomycin, and imipenem; reported resistance to trimethoprim-sulfamethoxazole	(53)
*Gordonibacter faecis* sp. nov.	*Eggerthellaceae*	Feces from a healthy subject in the Republic of Korea	Not established	Anaerobic, motile, catalase-positive, oxidase-negative, non-spore-forming gram-positive bacillus; pale beige, semi-translucent, slightly convex colonies on Gifu anaerobic medium supplemented with 1% arginine-hydrochloride; optimal growth at 37°C; converts ellagic acid into urolithin; positive for D-fructose, D-glucose-6-phosphate, leucyl glycine arylamidase, inosine, L-fucose, L-methionine, L-phenylalanine, L-rhamnose, L-valine, and palatinose; negative for adonitol, D-arabitol, D-cellobiose, gentiobiose, glycerol, itaconic acid, L-glutamic acid, and propionic acid	(54)^*g*^
*Hominibacterium faecale* gen. nov., sp. nov.	No current family assignment	Isolates (2) from feces of healthy individuals in China and Russia	Not established	Anaerobic, non-motile, non-spore-forming gram-positive bacillus; isolate propagated on peptone/yeast extract basal medium supplemented with 20 mM L-arginine; optimal growth at 35°C; positive for L-arginine and γ-aminobutyrate; weakly positive for inulin, succinate, pyruvate, aspartate, serine, glutamine, L-proline, L-isoleucine, L-histidine, and L-phenylalanine	(55)^*f*^
*Mobiluncus massiliensis* sp. nov.	*Actinomycetaceae*	Vaginal sample from a pregnant patient in France	Not established	Anaerobic, motile, catalase-negative, oxidase-negative, non-spore-forming gram-variable bacillus; 2- to 3 mm diameter circular, colorless, translucent, convex colonies on Columbia agar with 5% sheep blood; optimal growth at 37°C; positive for acid phosphatase, α-glucosidase, β-glucosidase, D-glucose, and D-mannose; negative for alkaline phosphatase, naphthol-AS-BI-phosphohydrolase, and valine arylamidase; reported MIC of <0.16 µg/mL to penicillin, 0.002 µg/mL to imipenem, <0.016 µg/mL to clindamycin, 0.125 µg/mL to vancomycin, and 0.25 µg/mL to tobramycin; ciprofloxacin and metronidazole demonstrated no activity	(56)^*c*^
*Neglectibacter timonensis* gen. nov., sp. nov.	*Oscillospiraceae*	Feces from a woman with type 2 diabetes	Not established	Initial characterization found in reference 57; anaerobic, catalase-negative, oxidase-negative, non-spore-forming gram-positive bacillus; 0.5 mm diameter circular, yellowish colonies on Columbia agar supplemented with 5% sheep blood; optimal growth at 37°C; positive for α-galactosidase, β-galactosidase, α-glucosidase, and β-glucosidase; negative for alkaline phosphatase, leucine arylamidase, α-fucosidase, glycerol, D-glucose, D-maltose, and D-saccharose	(58)^*c*^
*Peptoniphilus* *genitalis* sp. nov.	*Peptoniphilaceae*	Vaginal sample from a patient with recurrent miscarriages in France	Not established	Anaerobic, non-motile, catalase-negative, oxidase-negative, non-spore-forming gram-positive coccus; 2- to 3 mm diameter circular, gray, opaque, convex colonies on Columbia agar with 5% sheep blood; optimal growth at 37°C; positive for acid phosphatase, naphthol-AS-BI-phosphohydrolase, and D-glucose; negative for alkaline phosphatase, valine arylamidase, α-glucosidase, β-glucosidase, and D-mannose; reported MIC of <0.016 µg/mL to penicillin, 0.004 µg/mL to imipenem, 1.5 µg/mL to ciprofloxacin, 0.5 µg/mL to clindamycin, 0.5 µg/mL to metronidazole, 0.064 µg/mL to vancomycin, and 24 µg/mL to tobramycin	(56)^*c*^
*Pilosibacter fragilis* gen. nov., sp. nov.	*Clostridiaceae*	Feces from a healthy donor in China	Not established	Anaerobic, motile gram-positive bacillus; 3- to 5 mm diameter smooth, wet, translucent, circular, entire colonies on modified Gifu anaerobic medium; optimal growth at 37°C; positive for C4 esterase, cellobiose, acid phosphatase, and C8 esterase; negative for alkaline phosphatase, α-galactosidase, β-galactosidase, β-glucosidase, and β-glucuronidase	(49)
Gram-negative anaerobes					
*Faecalibacterium taiwanense* sp. nov.	*Oscillospiraceae*	Isolates (2) from feces of healthy adults in Taiwan	Not established	Anaerobic, non-motile, catalase-negative, non-spore-forming gram-negative bacillus; 0.5- to 1.5 mm diameter circular, yellowish, convex, entire colonies on supplemented BHI agar; growth at 30°C–45°C; unable to grow in 2% oxgall; positive for leucine arylamidase, acid phosphatase, β-glucosidase, mannose, raffinose, tyrosine arylamidase, alanine arylamidase, and serine arylamidase; negative for α-glucosidase, β-glucuronidase, leucyl glycine arylamidase, glycine arylamidase, D-malic acid, and glyoxylic acid	(59)
*Porphyromonas vaginalis* sp. nov.	*Porphyromonadaceae*	Vaginal sample from a patient in France	Not established	Anaerobic, non-motile, oxidase-negative, catalase-negative, non-spore-forming gram-negative bacillus; 1- to 2 mm diameter circular, black, smooth, convex colonies on Columbia agar with 5% sheep blood; optimal growth temperature at 37°C; positive for alkaline phosphatase, acid phosphatase, naphthol-AS-BI-phosphohydrolase, and D-glucose; negative for leucine arylamidase, D-mannose, D-sucrose, α-glucosidase, β-glucosidase, and β-glucuronidase; reported MIC of <0.016 µg/mL to penicillin, 0.016 µg/mL to imipenem, 1 µg/mL to ciprofloxacin, 0.023 µg/mL to clindamycin, 0.38 µg/mL to metronidazole, 2 µg/mL to vancomycin	(60)^*c*^
*Selenobaculum gibii* gen. nov., sp. nov.	*Selenomonadaceae*	Feces from an adult with Crohn’s disease in South Korea	Not established	Originally published as *Selenobaculum gbiensis*; anaerobic, motile, oxidase-negative, catalase-negative, non-spore-forming gram-negative bacillus; <1 mm diameter creamy, circular colonies on modified Gifu anaerobic medium; optimal growth at 26°C–37°C; positive for alkaline phosphatase, C8 esterase lipase, leucine arylamidase, glycerol, and D-adonitol; negative for α-galactosidase, β-galactosidase, α-glucosidase, β-glucosidase, *N*-acetyl-β-glucosaminidase, D-arabinose, L-arabinose, D-xylose, D-galactose, dulcitol, inositol, amygdalin, arbutin, D-cellobiose, D-tagatose, and L-fucose	(61)^*e*^
Anaerobic bacterium not characterized by Gram reaction					
*Methanomethylophilus alvi* gen. nov., sp. nov.	*Methanomethylophilaceae*	Feces from an elderly subject	Not established	Phenotypic characterization not provided	(62)^*b*^

^*a*^ Number of isolates from clinical source is 1, except where shown in parentheses.

^*b*^ Validly published by inclusion in Validation List no. 216 (18).

^*c*^ Validly published by inclusion in Validation List no. 219 (63).

^*d*^ Validly published by inclusion in Validation List no. 220 (64).

^*e*^ Validly published by inclusion in Validation List no. 217 (65).

^*f*^ Validly published by inclusion in Validation List no. 215 (66).

^*g*^ Validly published by inclusion in Validation List no. 218 (67).

^*h*^ BHI, brain heart infusion: MALDI-TOF MS, matrix-assisted laser desorption/ionization time-of-flight mass spectrometry; MIC, minimum inhibitory concentration.

**TABLE 2:** Revised bacterial taxa relative to human clinical material accepted from January 2024 to December 2024

Earlier validly published name	2024 validly published name	Other information	References
Gram-positive cocci			
*Kocuria polaris*	*Kocuria rosea* subsp. *polaris* comb. nov.	Initial description of the *K. polaris* taxon from an environmental source found in reference 68, incidence in duodenal mucosa documented in reference 69	(70)
*Kocuria rosea*	*Kocuria rosea* subsp. *rosea* comb. nov.	Subset of *K. rosea* strains revised to *Kocuria rosea* subsp. *rosea* comb. nov.; *K. rosea* remains a valid and correct taxon; examples of *K. rosea* involvement in human disease found in references 71 and 72	(70)
Gram-positive bacilli			
*Bacillus badius*	*Pseudobacillus badius* comb. nov.	Initial description of the *B. badius* taxon from a pediatric intestinal source found in reference 73 and accepted (74)	(75)^*a*^
*Lactobacillus delbrueckii* subsp. *sunkii*	*Lactobacillus delbrueckii* subsp. *allosunkii* nom. nov.	Initial description of the *L. delbrueckii* subsp. *sunkii* taxon found in reference 76, report of bacteremia found in reference 77, report of lung microbiome constituency found in reference 78	(79)
Gram-negative coccus			
*Catenococcus thiocycli*	*Allocatenococcus thiocycli* comb. nov.	Initial description of the *C. thiocycli* taxon found in reference 80, report of constituency in human nasal microbiome found in reference 81	(82)
Gram-positive anaerobes			
*Actinomyces oralis*	*Actinomyces acetigenes* sp. nov.	initial description of the *Actinomyces naeslundii* taxon found in reference 83 and accepted (74); *A. naeslundii* genospecies II, serotype III classification described in reference 84; taxonomic revision to *A. oralis* noted in reference 85	(86)^*b*^
*Actinomyces naeslundii*	*Actinomyces stomatis* sp. nov.	Initial description of the *Actinomyces naeslundii* taxon found in reference 83 and accepted (74), *A. naeslundii* strain described herein initially characterized in reference 87	(86)^*b*^
*Clostridioides mangenotii*	*Metaclostridioides mangenotii* comb. nov.	Taxonomic revision to *C. mangenottii* described in reference 88 and accepted (89); taxon also holds a *Clostridium mangenotii* synonym	(53)
Gram-negative anaerobes			
*Brotolimicola acetigignens*	*Waltera acetigignens* comb. nov.	Initial description of the *B. acetigignens* taxon from human feces found in reference 90 and accepted (91)	(92)
*Fournierella massiliensis*	*Allofournierella massiliensis* comb. nov.	Initial description of the *F. massiliensis* taxon from human feces found in reference 93	(82)
Curved bacterium			
*Butyrivibrio crossotus*	*Eshraghiella crossota* comb. nov.	Initial description of the *B. crossotus* taxon found in reference 94 and accepted (74), report of gut microbiome constituency found in reference 95	(96)
Obligate intracellular bacterium			
*Chlamydophila caviae*	*Chlamydia caviae* comb. nov	Initial valid publication of the *Chlamydophila caviae* taxon found in reference 97, incidence in community-acquired pneumonia documented in reference 98	(99)^*c*^

^*a*^ Validly published by inclusion in Validation List no. 220 (64).

^*b*^ Validly published by inclusion in Validation List no. 217 (65).

^*c*^ Validly published by inclusion in Validation List no. 218 (67).

**TABLE 3:** Update on clinical relevance for selected novel taxa described in the *JCM^a^* taxonomy compendia published in 2017, 2019, 2021, 2023, and 2024

Taxon	*JCM* compendium	Human source initially reported	Example(s) of clinical update	References
*Butyricimonas paravirosa*	2017 (27)	Feces	Bacteremia in a patient with acute terminal ileitis in the United States	(100)
*Lachnoanaerobaculum orale*	2017 (27)	Saliva from a healthy man	Bacteremia in a patient with acute lymphocytic leukemia in Japan following chemotherapy, bacteremia in a patient with acute myeloid leukemia and stomatitis in Canada following chemotherapy	(101, 102)
*Lachnoanaerobaculum* *umeaense*	2017 (27)	Small intestine of a child with celiac disease	Bacteremia in a patient with acute myeloid leukemia in France	(103)
*Dielma fastidiosa*	2019 (28)	Feces	Bacteremia in a hospitalized patient with diverticulitis	(104)
*Gardnerella leopoldii*	2021 (29)	Vaginal swab	Increased prevalence in patients with bacterial vaginosis	(105)
*Gardnerella piottii*	2021 (29)	Vaginal swab	Increased prevalence in patients with bacterial vaginosis	(105)
*Gardnerella swidsinskii*	2021 (29)	Vaginal swab	Increased prevalence in patients with bacterial vaginosis	(105)
*Chimaeribacter arupi*	2023 (30)	Wound, blood, and lower respiratory tract isolates submitted to reference laboratory	Bacteremia in a neonate in Germany and abdominal sepsis in a patient in Argentina	(106, 107)
*Eikenella halliae*	2023 (30)	Ocular swab and maxillary sinus archival isolates	Empyema post-pulmonary surgery in a patient in China	(108)
*Pandoraea commovens*	2023 (30)	Not specified (cystic fibrosis patients)	Outbreak within non-cystic fibrosis patients at a university hospital and satellite heart center in Germany	(109)
*Gardnerella greenwoodii*	2024 (5)	Urine	Increased prevalence in patients with bacterial vaginosis	(105)
*Gardnerella pickettii*	2024 (5)	Urine	Increased prevalence in patients with bacterial vaginosis	(105)
*Ottowia massiliensis*	2024 (5)	Feces	Canaliculitis in a patient in China	(110)

^*a*^ *JCM*, *Journal of Clinical Microbiology*.

### Novel taxa

Several novel gram-positive cocci belonging to the genera *Dolosigranulum*, *Staphylococcus*, and *Streptococcus* have recently been described. *Staphylococcus brunensis* sp. nov., a *Staphylococcus* sp. other than *S. aureus* (SOSA), was recovered from human clinical material as reported by Kovařovic et al*.* (32). In at least one instance, isolation was associated with inflammation (32). This organism is most closely related to the *Staphylococcus petrasii* complex. Matrix-assisted laser desorption/ionization time-of-flight mass spectrometry (MALDI-TOF MS) profiles of the five recovered isolates were unique compared to other SOSA. Disk diffusion susceptibility testing using European Committee on Antimicrobial Susceptibility Testing (EUCAST) guidelines for interpretation revealed resistance to penicillin (*blaZ* genotype) and erythromycin (*ermC* genotype), and susceptibility to oxacillin, cefoxitin, clindamycin, trimethoprim/sulfamethoxazole, linezolid, and tetracycline (32).

The pathogenic potential of several of the newer streptococci (Table 1) is not clear, as limited clinical data were provided. One exception is *Streptococcus suis* subsp. *hashimotonensis* subsp. nov., which was recovered from a wound infection following a wild boar bite (36). The patient experienced fever, chills, and purulent drainage from the bite wound. The culture of the wound grew an organism identified as *S. suis*. However, unlike *S. suis*, this isolate possessed Lancefield group A antigen and had additional peaks when tested using MALDI-TOF MS (36). This new subspecies was also recovered from oral swabs of wild boars living in the same geographic location in Japan (111).

Previously believed to be a sterile fluid, it is now known that breast milk has a unique microbiota with streptococci being the dominant constituents (33). Three novel taxa, *Streptococcus hohhotensis* sp. nov., *Streptococcus raffinosi* sp. nov., and *Streptococcus humanilactis* sp. nov., were recovered from breast milk of otherwise healthy nursing mothers in China and Vietnam (33–35).

Among the novel gram-positive bacilli, two organisms may have importance with respect to human disease. *Cellulomonas endometrii* sp. nov. was recovered from a French woman who suffered from chronic endometritis resulting in recurrent early miscarriages (38). The authors rightly raise the question as to whether this facultative anaerobe contributed to the patient’s endometritis. Two isolates recovered from separate patients with pulmonary infections in China and most closely related to *Nocardia beijingensis* were named *Nocardia implantans* sp. nov. (40). Notably, these isolates were multidrug resistant, with susceptibility only to imipenem and cefepime. Trimethoprim-sulfamethoxazole susceptibility was not assessed.

Four novel members of order *Enterobacterales* were included on Validation Lists or published in 2024, distributed among families *Enterobacteriaceae*, *Morganellaceae*, and *Yersiniaceae* (112). Parenthetically, in 2024, the clinical and veterinary microbiology disciplines were recipients of an eighth included *Enterobacterales* family designation, namely, *Gallaecimonadaceae* fam. nov. (66, 113). In addition to the *E. pasteurii* that was introduced in an earlier section of this report, *Enterobacter pseudoroggenkampii* sp. nov. (42) was effectively published and subsequently included on a Validation List; however, evidence of clinical significance was provided. Over the course of 7 months, two patients in China were hospitalized with pneumonia or purulent diabetic foot ulcers. The isolated *E. pseudoroggenkampii* could be differentiated from other *Enterobacter* spp. by its gelatinase activity and inability to metabolize L-rhamnose. *E. pseudoroggenkampii* exhibited several phenotypic susceptibility traits that are common to other *Enterobacter* spp.: resistance to ampicillin, ampicillin-sulbactam, cefazolin, and cefuroxime. Reduced susceptibility to fluoroquinolone agents was also documented. In contrast, the novel *E. pasteurii*, derived from an archival collection in France (20), was susceptible to ampicillin, piperacillin, cephalothin, ceftazidime, and ciprofloxacin.

*Providencia huashanensis* sp. nov. (45) was initially identified in China within the realm of healthcare-associated infection. One patient underwent laparoscopic exploration for persistent dull upper abdominal pain, was diagnosed with acute diffuse peritonitis, and sustained an incision infection approximately 10 days later. The patient improved on a broad-spectrum regimen (cefoperazone-sulbactam, imipenem, metronidazole, piperacillin-tazobactam, and moxifloxacin) designed to manage the peritonitis. A second patient at a different hospital was diagnosed with a urinary tract infection secondary to a hemostasis and fluid replacement intervention for cerebral infarction. Sequential piperacillin-tazobactam, amikacin, and cefoperazone managed the infection until transfer for end-of-life care. A third patient at the same hospital underwent surgery for removal of an intracranial hematoma and subsequently developed an intracranial infection with *P. huashanensis*. Concomitant to the surgery, the patient had several areas of density in the right lung. The clinical course included administration of cefuroxime, cefoperazone-sulbactam, and meropenem. All three isolates were initially identified as *Providencia rettgeri* by MALDI-TOF MS; however, these novel isolates were negative for indole production and gelatinase. 16S rRNA gene and housekeeping gene (*n* = 5) phylogenetic reconstruction revealed that the three isolates were clonal. Furthermore, acquired genes conferring carbapenemase production, plasmid-mediated quinolone resistance, extended-spectrum β-lactamase production, and aminoglycoside-modifying enzymes were identified in each isolate.

Four total isolates of *Serratia sarumanii* sp. nov. were reported by Klages et al. (46). The organism was implicated in three skin and soft tissue infections due to corresponding elevated C-reactive protein values and/or elevated peripheral leukocyte counts. A fourth isolate was cultivated in a urine specimen from a patient with elevated positive acute-phase reactants. The authors did note the concomitant isolation of *Pseudomonas aeruginosa* and *Klebsiella oxytoca* from select wound and urine cultures, respectively. The type strain of *S. sarumanii* added piperacillin, piperacillin-tazobactam, cefotaxime, and ceftazidime resistance to traditional phenotypes of ampicillin resistance noted for other clinically relevant *Serratia* spp.

Two genera that house novel non-glucose-fermentative Gram-negative bacilli merit discussion. In addition to the clinical significance of *Ralstonia insidiosa* (114), *Ralstonia mannitolilytica* (115), and *Ralstonia pickettii* (116) in human medicine (particularly as these microbes pertain to cystic fibrosis), Steyaert et al. (21) posit the potential importance of *Ralstonia flaminis* sp. nov., *Ralstonia flatus* sp. nov., *Ralstonia holmesii* sp. nov., and *Ralstonia thomasii* sp. nov. Archival and historic collections comprised the source material for characterization of the four novel taxa. Isolates were multinational in nature and derived from sputum and blood, and from at least one patient with status post-tracheotomy. Other strains within these *Ralstonia* spp. (other than *R. flaminis*) were derived from aquatic and even agricultural reservoirs. Isolates of *R. flaminis*, *R. flatus*, and *R. thomasii* were specifically described as emanating from cystic fibrosis patients. Additional clinical information, including potential co-isolation of other pathogens, was not provided.

*Stenotrophomonas forensis* sp. nov. (species name derived from the Latin *forensis*, meaning public) was published by scientists at the District of Columbia (United States) public health laboratory. In the wake of large-scale SARS-CoV-2 outreach testing in 2020, contaminated virus transport medium tubes were not uncommon (117). From one such tube, Nguyen et al. (47) cultivated a gram-negative bacillus on blood agar that was identified as *Stenotrophomonas maltophilia* via MALDI-TOF MS (probable species identification). Subsequent sequencing indicated that the isolate was not a member of the *S. maltophilia sensu stricto* genomospecies, but rather a unique taxon. Subsequent *in silico* analysis of 26 National Center for Biotechnology Information GenBank sequences that were deposited under the auspices of *S. maltophilia* revealed homology to *S. forensis*. Dates of antecedent isolation ranged from 1999 to 2023, with continents of origin including North America, South America, Europe, Asia, and Australia. Isolates have been documented from humans, horses, and avian species.

A second *Stenotrophomonas* spp., *Stenotrophomonas pigmentata* sp. nov. (48), with its characteristic brown-pigmented colony, was isolated in China from a patient with pulmonary tuberculosis. Although the anatomic source of isolation was not provided in the publication, putative pathogenicity was ascertained in a murine model, manifested by weight loss and pulmonary congestion. Cytokine profiling in infected mice resembled patterns found in patients with tuberculosis. *S. pigmentata* resistance to trimethoprim-sulfamethoxazole, ampicillin, nalidixic acid, ciprofloxacin, ampicillin-sulbactam, meropenem, ceftazidime-avibactam, ceftazidime, and cefotaxime was claimed by the authors, though a CLSI document for mycobacteria and aerobic actinomycetes was referenced for interpretation.

*Kingella pumchi* sp. nov. was initially isolated from vertebral puncture tissue of a patient in China (43). No additional clinical information was presented. This short, oxidase-positive, catalase-negative gram-negative bacillus was cultivated on blood agar. *K. pumchi* can be phenotypically differentiated from *Kingella kingae* by its inability to metabolize fructose, maltose, and sucrose and lack of alkaline phosphatase production. No significant antimicrobial resistance genotype was detected.

A novel *Bartonella* spp. initially published in 2008 (41) was included in Validation List no. 220 in 2024 (64). That primary publication is further noteworthy because it documented the first culture-confirmed *Bartonella* spp. infection of humans in Thailand. *Bartonella tamiae* sp. nov. was recovered from three patients (two of whom were febrile at the time of presentation; one presented with ocular disease) who reported killing or trapping rats in their homes. Two of the patients recalled potential exposure within the previous 2 weeks. Mild anemia (presumed infection of erythrocytes), headache, myalgia, and liver function abnormalities were noted in all patients. The bacterium was recovered from blood clots by either (i) co-cultivation with Vero E6 cells for 7 days, then subculture onto rabbit blood-enriched agar or (ii) inoculation into liquid *Bartonella-Alphaproteobacteria* growth medium for 7 days, followed by subculture onto rabbit blood agar. Differentiation from other *Bartonella* spp. was ascertained by molecular analysis of culture contents and off-line analysis of biochemical properties.

Of the 15 novel obligate anaerobic taxa (11 gram positive, 3 gram negative, and 1 not characterized via Gram morphology) listed throughout Table 1, 12 were originally isolated from fecal specimens. The vast majority of these individuals were described as healthy subjects and/or contributors to various microbiome studies. Possible exceptions to this paradigm include the recovery of *Faecalimicrobium dakarense* gen. nov., sp. nov. from a child with gastroenteritis (53), *Neglectibacter timonensis* gen. nov., sp. nov. from a diabetic adult (58), and *Selenobaculum gibii* gen. nov., sp. nov. from a patient with Crohn’s disease (61). However, data on clinical course, ancillary microbiology studies (e.g., virus culture and target-specific nucleic acid amplification testing), or non-infectious causes of these maladies were not provided. Two studies reported three novel taxa in the context of vaginal microbiota. Abou Chacra et al. (56) characterized *Peptoniphilus genitalis* sp. nov. that was recovered from a patient who had endured multiple miscarriages. In the same publication, the Gram-variable bacillus *Mobiluncus massiliensis* sp. nov. was identified from vaginal sampling of a patient at 26 weeks of gestation. The authors noted no evidence of bacterial vaginosis or a sexually transmitted infection in either patient. An earlier report of several dozen bacterial isolations from a vaginal swab collected from a non-pregnant woman (60) included that of *Porphyromonas vaginalis* sp. nov.

Of further interest are two Validation List entries throughout 2024 (18, 64) that have since reverted to synonym status and are not included in Table 1. *Corynebacterium ramonii* sp. nov. (18, 118), a member of the *Corynebacterium diphtheriae* species complex, was discovered during phenotypic and genotypic characterization of a collection of *Corynebacterium ulcerans* lineages 1 and 2. The isolates were recovered from cutaneous, respiratory, and invasive sources and housed in a repository at the French National Reference Center (118). The authors determined that *C. ulcerans* lineage 2 was sufficiently different from isolates of lineage 1 and named the novel species *C. ramonii*. Unlike *C. ulcerans*, which is primarily a zoonosis, *C. ramonii* isolates were capable of human-to-human transmission. Many of these isolates were also toxigenic (118).

Isolates of *Borrelia hermsii* have historically been divided into clades and designated as *B. hermsii* genomic groups I and II by way of multilocus sequence typing. Schwan et al. (119) sequenced 11 *B. hermsii* isolates across the two genomic groups and reported DNA sequence identity of 95.86%–95.99% (chromosomal lengths of approximately 920,000 bp) between representatives of the two genomic groups. Isolates in genomic group II were granted taxonomic status of *Borrelia nietonii* sp. nov. (119), as included on Validation List no. 220 (64), for delineation from *Borrelia hermsii sensu stricto*. Isolates of *B. nietonii* were previously described from human (120) and *Ornithodoros hermsi* (121) sources.

### Taxonomic revisions

*Kocuria* spp. are gram-positive cocci that survive in diverse environments from mammalian skin to marine sediments (70). Using comprehensive phylogenetic analyses, Ghodhbane-Gtari et al. refined the taxonomic status of some *Kocuria* spp. The authors propose that *Kocuria polaris* is a subspecies of *Kocuria rosea* and that *Kocuria rosea* be revised to *Kocuria rosea* subsp. *rosea* comb. nov. The latter organisms have caused serious infections such as bacteremia complicating SARS-CoV-2 infection (71) and infectious endocarditis (72). Two gram-positive bacilli have undergone taxonomic revision (Table 2). *Bacillus badius* (proposed by Verma et al. [75] as *Pseudobacillus badius* comb. nov.) is one of the oldest members of the *Bacillus* genus. It was originally described as a spore-forming bacillus recovered from the feces of children (73). An environmental organism found in water and vegetative matter, it has not been associated with human infection. *Lactobacillus delbrueckii* subsp. *sunkii*, now proposed as *Lactobacillus delbrueckii* subsp. *allosunkii* comb. nov., was originally isolated from a Japanese pickle (76) and is an opportunistic pathogen, as described by Macho-Aizpurua et al. (77). In that paper, the authors present an elderly immunocompromised patient with bacteremia related to a urinary tract infection caused by *L. delbrueckii* subsp. *sunkii*.

The gram-negative coccus *Catenococcus thiocycli* was initially reported in 1992 from an environmental source in Papua New Guinea (80). Thirty years later, a report (81) associated an increased abundance of this organism, among others, with increased skin disease burden in the context of cutaneous T-cell lymphoma. The genus *Catenococcus* has subsequently been declared illegitimate, with deferral to the novel genus *Allocatenococcus* (82).

In general, taxonomic revisions of obligate anaerobic bacteria involved organisms that are commensals of the human gastrointestinal tract. Three additional changes will be discussed. The proposals of *Actinomyces acetigenes* sp. nov. and *Actinomyces stomatis* sp. nov. (86) go back to an effective description of *Actinomyces naeslundii* from the human oral cavity in 1951. Two subsequent genospecies within *A. naeslundii* (84) gave rise to additional serotype classifications within each genospecies. Tian et al. (86) used whole genome sequencing to clarify intermediate taxonomic classifications such as *A. naeslundii* and *Actinomyces oris* into the species designations listed in Table 2. Of the two, *A. acetigenes* has been implicated in progressive periodontitis. As if the transfer of two species of *Clostridium* to novel genus *Clostridioides* were not enough in 2016 (particularly in the realm of infection prevention and healthcare-associated infection) (88, 89), one of those taxa (*Clostridioides mangenotti*) has now been assigned to the novel genus *Metaclostridioides* (53).

The taxon *Chlamydophila caviae* was effectively and validly published in 1999, with initial recovery from conjunctiva of guinea pigs (97). The publication of Everett et al. also marked the reassignment of past *Chlamydia* spp. (such as *Chlamydia felis*, *Chlamydia pecorum*, *Chlamydia pneumoniae*, and *Chlamydia psittaci*) as members of genus *Chlamydophila*. The year 2015 marked the proposal of Sachse et al. to return these four species to genus *Chlamydia* (99), along with proposed reclassification of *C. caviae* and *Chlamydophila abortus* as species of *Chlamydia*. The revision of *C. caviae* was included on a Validation List in 2024 (Table 2).

### Recently ascribed and additional clinical significance

The obligate anaerobic gram-negative bacilli of intestinal origin *Butyricimonas paravirosa* and *Dielma fastidiosa* were initially discussed in *Journal of Clinical Microbiology* compendia dating back to 2017 (Table 3). Within the past year, literature has entertained the clinical significance of these two species in bacteremia with gastrointestinal comorbidities. With respect to *B. paravirosa*, a febrile patient with acute terminal ileitis yielded the organism in anaerobic bottles of two sets of blood cultures and responded to a 7-day course of ceftriaxone and metronidazole (100). In Denmark, *D. fastidiosa* was isolated within day 2 blood cultures from a patient that was diagnosed with extensive diverticulitis in the descending colon (104). This anaerobic bacterium was interpreted by EUCAST breakpoints to be susceptible to piperacillin-tazobactam, penicillin, metronidazole, and clindamycin. Blood cultures collected on days 5 and 6 yielded *Escherichia coli* and *Parabacteroides distasonis*, respectively. The patient was treated with a 7-day course of piperacillin-tazobactam, was discharged, then was readmitted with recurrent fever and abdominal pain. Treatment success was attained with four additional days of piperacillin-tazobactam, followed by ciprofloxacin and metronidazole. Overall clinical course and additional blood culture isolates cast doubt on the clinical significance of *D. fastidiosa*.

Three recent reports of patients with hematologic malignancy highlight potential roles for *Lachnoanaerobaculum* spp. in clinical disease. An elderly patient with acute myeloid leukemia presented to a French hospital with fever, gingival pain, weight loss, and loss of appetite (103). A drug-induced anemia and aplasia was initiated 10 days previously, managed in part by trimethoprim-sulfamethoxazole. *Lachnoanaerobaculum umeanse* was recovered from anaerobic blood cultures after 36 hours. The clinical course of the patient improved briefly upon treatment with amoxicillin-clavulanic acid and ciprofloxacin (despite increased ciprofloxacin minimum inhibitory concentration [MIC] value) until the patient succumbed to invasive aspergillosis. A pair of reports has described isolations of *Lachnoanaerobaculum orale* in clinical settings. Ida et al. (101) reported a case of neutropenic fever with severe stomatitis after 15 days of chemotherapy in a Japanese patient with acute lymphoblastic anemia. Organisms were detected in an anaerobic blood culture bottle at 33 hours of incubation. *L. orale* was subsequently shown to yield low MIC for penicillin, cefepime, and meropenem, with elevated MIC for moxifloxacin, levofloxacin, and ciprofloxacin. Initial empiric therapy of ciprofloxacin and cefepime was converted to doripenem, with patient improvement. Sabrie et al. (102) described an *L. orale* bacteremia in a Canadian patient with chemotherapy-induced neutropenia for acute myeloid leukemia. Comorbidities at the time included hepatitis B infection and latent tuberculosis infection, both of which indicated appropriate therapy. The patient presented with fever, severe stomatitis, and digit edema. *L. orale* was isolated from one anaerobic blood culture bottle. Additional culture studies were non-contributory. The patient was treated with ciprofloxacin and amoxicillin-clavulanic acid for febrile neutropenia and bacteremia. Ciprofloxacin susceptibility testing data were not procured.

Two past publications have shared five novel species within the *Gardnerella* genus (122, 123), in addition to *Gardnerella vaginalis*. In both point prevalence and prospective studies, Munch et al. (105) determined that prevalence and burden of *Gardnerella greenwoodii*/*Gardnerella swidsinskii*, *Gardnerella leopoldii*, and *Gardnerella picketii*/*Gardnerella piottii* groupings in vaginal swabs were each statistically increased in cases of bacterial vaginosis (detection frequency ranging from 64.4% to 91.1%) than in patients not experiencing the condition. Similar findings were made for *G. vaginalis*. Prevalence of these groupings in patients without bacterial vaginosis ranged from 25.3% (*G. leopoldii*) to 44.0% (*G. picketii*/*G. piottii*). Interestingly, the authors noted that the presence of three or more groupings of *Gardnerella* spp. (*G. vaginalis* was included in this assessment) in patients without bacterial vaginosis was more predictive of patients returning within 100 days with bacterial vaginosis than in patients with fewer than three groupings of *Gardnerella* spp. No single *Gardnerella* spp. was a specific marker for bacterial vaginosis.

*Chimaeribacter arupi* was one of three species of the novel gram-negative bacillus genus *Chimaeribacter* initially elucidated from wound, lower respiratory tract, and blood specimens that were submitted to a US reference laboratory for definitive identification (124). As such, limited (if any) clinical data were linked to these isolates. Riediger et al. (106) described isolation of this species from a tunneled central venous catheter instilled in a 19-month-old female in Germany who presented for emergent care with fever and somnolence. Due to birth complications, the patient underwent resection of portions of the ileum, jejunum, ascending colon with cecum, and right colic flexure, resulting in short bowel syndrome. The clinical course was further complicated by multiple hospitalizations due to catheter infections, nutritional challenges, and recent diagnosis of Crohn’s disease. *C. arupi* was recovered from a pediatric blood culture bottle and was susceptible to all antimicrobial agents tested with the exception of ampicillin (fosfomycin MIC was >128 mg/L; no EUCAST or CLSI interpretive criteria for this agent). The patient was treated with meropenem for 14 days to prevent potential biofilm formation on the catheter device. Fever and acute-phase reactant values decreased upon antimicrobial therapy. In a second case, Barberis et al. (107) isolated *C. arupi* from an Argentinian patient with a history of rectal adenocarcinoma treated with chemotherapy. The current presentation involved fever and abdominal pain. The peritoneal fluid isolate grew within 24 hours on blood agar and Levine eosin methylene blue agar and was susceptible to all agents tested with the aforementioned ampicillin and fosfomycin caveats. Organism identification was made by genome sequencing of the isolate, as methods such as MALDI-TOF MS and 16S rRNA sequencing proved inconclusive. The patient was discharged after 7 days of ceftriaxone therapy.

Genomic sequencing was applied in an alternative fashion for a recent report of *Eikenella halliae* infection. Huang et al. (108) presented a Chinese patient with lung cancer that underwent radical lung resection 3 weeks previously and was currently admitted for a 3-day history of fever, cough, and expectoration. Colonial growth from a pleural effusion specimen was identified to genus-level *Eikenella* by way of 16S rRNA gene sequencing. Scientists then turned to direct metagenomic next-generation sequencing on two additional sputum specimens. *E. halliae* was identified from one of the two specimens. The authors cite the pathogenicity and future importance of *E. halliae* in the management of empyema in immunocompromised patients.

In an initial description of *Pandoraea commovens* in 2019, Peeters et al. (125) reported that the bacterium was derived from cystic fibrosis patients. A German group (109) reported a 3-year outbreak of the organism in 24 non-cystic fibrosis patients who required intensive care. Common features among the patients included critical illness, mechanical ventilation, surgical intervention, and previous antimicrobial therapy. Immunocompromised or diabetic status was noted in a minority of patients. Ten of these isolates were judged to be associated with clinically significant infection. Four deaths within this cohort were independent of *P. commovens* infection. The majority of *P. commovens* isolates in this report were resistant to agents in several antimicrobial classes with the exception of ampicillin-sulbactam and imipenem. A second non-glucose-fermentative gram-negative bacillus, *Ottowia massiliensis*, was recovered in China from a lacrimal duct secretion from a woman with a 4-year history of bilateral epiphora and discharge that worsened prior to presentation (110). Identification was made by 16S rDNA analysis of colonial growth. The infection was effectively treated with levofloxacin and pranoprofen eye drops. The authors of this case study caution microbiologists regarding potential deficits in reporting this pathogen, including its slow growth rate (approximately 5 days) and its absence from MALDI-TOF MS databases.

## CONCLUSION

Consistent with prior compendia, this review focused on novel species and revised taxa of relevance to human health and disease. Important information regarding disease potential of organisms included in prior reviews adds to knowledge regarding clinical relevance. Taxonomic revisions can also influence the procurement and interpretation of clinically relevant antimicrobial susceptibility testing (27, 126). It is recommended that medical microbiology laboratories consult and communicate with important stakeholders such as clinicians, infection control practitioners, public health colleagues, and pharmacists in the context of taxonomic revisions and new bacterial species discoveries. Published guidance documents and *Journal of Clinical Microbiology* compendia can assist in the implementation of necessary changes into routine microbiology operations and the timing of such implementation (7) to ultimately facilitate optimal patient care and properly impact public health.
